# OsBBX2 Delays Flowering by Repressing *Hd3a* Expression Under Long-Day Conditions in Rice

**DOI:** 10.3390/plants14010048

**Published:** 2024-12-27

**Authors:** Yusi Yang, Jiaming Wei, Xiaojie Tian, Changhua Liu, Xiufeng Li, Qingyun Bu

**Affiliations:** 1 College of Advanced Agriculture and Ecological Environment, Heilongjiang University, Harbin 150080, China; 2 State Key Laboratory of Black Soils Conservation and Utilization, Key Laboratory of Soybean Molecular Design Breeding, NortheastInstitute of Geography and Agroecology, Chinese Academy of Sciences, Harbin 150081, China; 3 University of Chinese Academy of Sciences, Beijing 100049, China

**Keywords:** rice, heading date, BBX2, Hd1, Hd3a

## Abstract

Members of the B-Box (BBX) family of proteins play crucial roles in the growth and development of rice. Here, we identified a rice BBX protein, Oryza sativa BBX2 (OsBBX2), which exhibits the highest expression in the root. The transcription of *OsBBX2* follows a diurnal rhythm under photoperiodic conditions, peaking at dawn. Functional analysis revealed that *OsBBX2* possesses transcriptional repression activity. The *BBX2* was overexpressed in the rice japonica cultivar Longjing 11 (LJ11), in which *Ghd7* and *PRR37* were non-functional or exhibited weak functionality. The overexpression of *OsBBX2* (*OsBBX2 OE*) resulted in a delayed heading date under a long-day (LD) condition, whereas the *bbx2* mutant exhibited flowering patterns similar to the wild type (WT). Additionally, transcripts of *Ehd1*, *Hd3a*, and *RFT1* were downregulated in the *OsBBX2 OE* lines under the LD condition. *OsBBX2* interacted with *Hd1* (*BBX18*), and the *bbx2 hd1* double mutant displayed a late flowering phenotype comparable to that of *hd1*. Furthermore, *OsBBX2* enhanced the transcriptional repression of *Hd3a* through its interaction with *Hd1*, as demonstrated in the protoplast-based assay. Taken together, these findings suggest that the *OsBBX2* delays flowering by interacting with *Hd1* and co-repressing *Hd3a* transcription.

The BBX family of proteins is highly conserved across green plants. They are characterized by one or two B-Box motifs in the N-terminal domain and a specific CCT (CONSTANS, CO-like, and TO) domain at the C-terminus [1]. They are involved in many plant regulatory networks, ranging from seedling photomorphogenesis to stress response [2]. In *Arabidopsis thaliana*, a total of 32 genes encode BBX proteins, while in rice, the BBX family comprises 30 proteins, designated as OsBBX1 to OsBBX30, based on their chromosomal locations. Among these, seven OsBBXs contain two B-box domains and a conserved CCT domain, ten possess one B-box and one CCT domain, three have a single B-box domain, and ten contain two B-box domains [1].

Most *BBX* family members have been reported to play a vital role in floral transition. *CONSTANS (CO)*/*BBX1* was the first *BBX* family member identified in *Arabidopsis*. *CO* induces flowering by activating the expression of *FLOWERING LOCUS T* (*FT*) and *SUPPRESSOR OF CONSTANS1* (*SOC1*) [3]. Subsequent studies have identified several BBX proteins that regulate flowering. For instance, *BBX7* (*COL9*) delays flowering under long-day (LD) conditions by reducing *CO* expression [4], while *bbx4* (*col3*) plants flower earlier during both short and long days, and *COL3* is a positive regulator of photomorphogenesis [5]. The late flowering phenotypes of overexpressed *BBX32* (*EIP6*) transgenic plants suggest that its interaction with *EMBRYONIC FLOWER1* (*EMF1*) can regulate flowering time in *Arabidopsis* [6]. *BBX19* functions as a negative regulator of flowering time, and it also functions as a regulator of circadian rhythm by complexing PRR proteins to enhance their repressive effect on *CCA1* transcription [7]. *BBX24* and *BBX6* (*COL5*) function as positive regulators of flowering, with *BBX24* overexpressing lines exhibiting accelerated flowering by striving for *FLOWERING LOCUS C* (*FLC*) to impact downstream flowering genes [8]. *BBX6* overexpression induces early flowering by promoting FT expression [9].

Rice *HEADING DATE 1* (*Hd1*/*OsBBX18*), which is an orthologue of *CO* in *Arabidopsis*, promotes heading under short-day (SD) conditions and inhibiting under long-day (LD) conditions [10]. In addition, a group of B-box-containing proteins were proven to repress flowering in rice. *BBX27* (*OsCO3*), which has two incomplete B-boxes, serves as a flowering repressor upstream of *Hd3a* under SD [11]. The overexpression of *OsBBX26* (*OsCOL15*) suppresses flowering by promoting *Ghd7* and repressing *RID1* under both SD and LD conditions [12]. The overexpression of *OsBBX17* (*OsCOL16*) delays heading under both SD and LD conditions by upregulating *Ghd7* transcripts [13]. *OsCOL13* functions as a negative regulator of flowering downstream of *OsphyB* and upstream of *Ehd1*. On the other hand, OsCOL13 is functionally redundant with BBX5 (OsCOL4), which is a constitutive flowering repressor upstream of *Ehd1* and downstream of *OsphyB* [14]. Both the overexpression of *BBX7* (*OsCOL9*) and *BBX10* (*OsCOL10*) delay flowering under SD and LD conditions by repressing *Ehd1*, *Hd3a*, and *RFT1* expression. OsCOL10 acts as a suppressor of rice flowering time by bridging *Ghd7* and *Ehd1* [15]. *OsBBX14* suppresses rice flowering by regulating either *Hd1* or *Ehd1* under LD or SD conditions [16].

Here, we show that *BBX2* acts as a negative regulator of flowering time under long-day (LD) conditions. *BBX2* physically interacts with *Hd1* to repress the transcription of *Hd3a*.

The *BBX2* (LOC_Os02g07930) gene was overexpressed in the rice *japonica* cultivar Longjing 11 (LJ11), resulting in two independent *BBX2* overexpressing (*OE)* transgenic lines, which exhibited significantly higher levels of *BBX2* expression (Supplemental Figure S1A). The *BBX2 OE* plants flowered outstandingly later than LJ11 under the LD (14 h light/10 h dark) condition, with delays of 9.35 and 10.65 days for the two approaches (Figure 1A,B). An RT-qPCR interpretation exhibited that the mRNA levels of *Ehd1*, *Hd3a*, and *RFT1* were dramatically lessened in the *BBX2 OE* plants compared to LJ11 (Supplemental Figure S1B–D). The decreased expression of the florigen genes and their catalyst *Ehd1* is consistent with the later flowering phenotypes observed in *BBX2 OE* plants (Figure 1A,B).

Most BBX proteins serve as flowering repressors [2]. To inspect the attributes of *BBX2*, we first assessed its transcriptional activity in a rice protoplast system; *BBX2* reduced *LUC* transcription activity compared with GAL4BD, revealing it to be a transcriptional repressor (Figure 1C,D). As BBX2 functions as a negative regulator of flowering, we investigated the diurnal expression modes of *BBX2* under LD and SD conditions. The *BBX2* manifestation began to accumulate slowly at dusk before peaking at dawn and sharply decreasing until dusk to its lowest level under both SD and LD conditions (Supplemental Figure S2A,B). In addition, we examined *BBX2*’s pattern of temporal and spatial expression in diverse tissues, including roots, stems, leaves, panicles, and seeds, by RT-qPCR. *BBX2* was shown mainly in the roots, with a comparative expression in the leaves, stem, and spike, and no expression was observed in the panicles or seeds (Supplemental Figure S2C).

To analyze the underlying mechanism for BBX2 in regulating flowering, we investigated the potential interacting proteins of BBX2 by the STRING database (https://cn.string-db.org/). We first used a yeast two-hybrid system to identify whether BBX2 could physically interact with Hd1, PRR1, and DTH2. The results demonstrated that BBX2 interacts with Hd1, but not with PRR1 or DTH2 (Figure 1E). To confirm this interaction, we applied split luciferase complementation assays by creating merger constructs of *BBX2* and *Hd1* to C- and N-terminal fragments of luciferase. *Agroinfiltration*-based transient assays in *N. benthamiana* showed the restoration of luciferase activity in the leaves co-infiltrated with BBX2 and Hd1 (Figure 1F).

To explore the genetic relationship between *BBX2* and *Hd1*, we generated *bbx2*, *hd1*, and *bbx2 hd1* mutants using CRISPR/Cas9-mediated genome editing in the LJ11 background [17]. Target sites were designed for the first exons of *BBX2* and *Hd1* (Supplemental Figure S3A,B). Despite the different mutation types in the single and double mutants, *BBX2* inserted a G base in the single mutant of *bbx2*, and a G base was missing in the *bbx2 hd1* double mutant. *Hd1* inserted a T base in the single mutant, and the A base was inserted in the *bbx2 hd1* double mutant, for which successful gene knockout was achieved (Supplemental Figure S3C,D). Phenotypic analysis showed that *bbx2* flowers at a similar time to LJ11 under the LD condition, while *hd1* and *bbx2 hd1* mutants flower approximately 5.1 to 5.6 days later than LJ11 under the LD condition (Figure 1G–J). These results suggest that Hd1 functions genetically downstream of BBX2. As a flowering repressor, Hd1 controls the expression of florigen genes under the LD condition. We hypothesized that BBX2 enhances the inhibitory effect on flowering by physically interacting with Hd1. Considering that BBX2 can interact with Hd1, which represses *Hd3a* expression, we performed a transient expression assay in rice protoplasts using *35S_Pro_:GFP* as a control and *35S_Pro_:BBX2*, *35S_Pro_:Hd1*, and *Hd3a_Pro_:LUC* as effectors and reporters, respectively (Figure 1K). The results indicate that *35S_Pro_:Hd1* efficiently suppressed *Hd3a* expression compared to the control *35S_Pro_:GFP*, and this suppression was further enhanced by the addition of *35S_Pro_:BBX2* (Figure 1L).

*Ehd1* and *Hd1* are key factors in regulating flowering in rice, particularly under long-day conditions [18]. It has been shown that Hd1 and Ghd7 interact to form a complex, which specifically binds to the cis-regulatory region of *Ehd1*, thereby suppressing its expression [19]. Additionally, studies have shown interactions between Hd1, Ghd7, and PRR37. Notably, Hd1 promotes flowering in both long- and short-day conditions in the *ghd7 prr37* double mutant [20]. This suggests that Hd1 functions in conjunction with Ghd7 and PRR37 under long-day conditions [21], while the *OsELF4s-OsELF3-1-OsLUX* complex reduces the expression of *Ghd7* and *PRR37* under short-day conditions. Therefore, Hd1 plays an independent role in the regulation of flowering [22]. Interestingly, it has been reported that *Ghd7* and *PRR37* are non-functional or exhibit weak functionality in the LJ11 background [23]. Therefore, knocking out *Hd1* in the LJ11 background results in a late flowering phenotype.

As *bbx2* flowered at a similar time to LJ11, this was probably due to functional redundancy with other *BBX* family members. To explore this, we investigated the evolutionary relationships within the *BBX* gene family. We identified *BBX21* as a homolog of *BBX2*; however, its role in regulating rice flowering remains unknown. Future research will involve creating a *bbx2 bbx21* double mutant to assess potential functional redundancy.

In summary, our study reveals that *BBX2* functions as a negative regulator of flowering, and the expressions of *Ehd1*, *Hd3a*, and *RFT1* are significantly reduced in *BBX2 OE* plants. *BBX2* exhibits transcriptional repression activity and can interact with Hd1. These results suggest that BBX2 interacts with Hd1, co-suppressing the expression of *Hd3a* and consequently delaying flowering in rice.

## Figures and Tables

**Figure 1:**
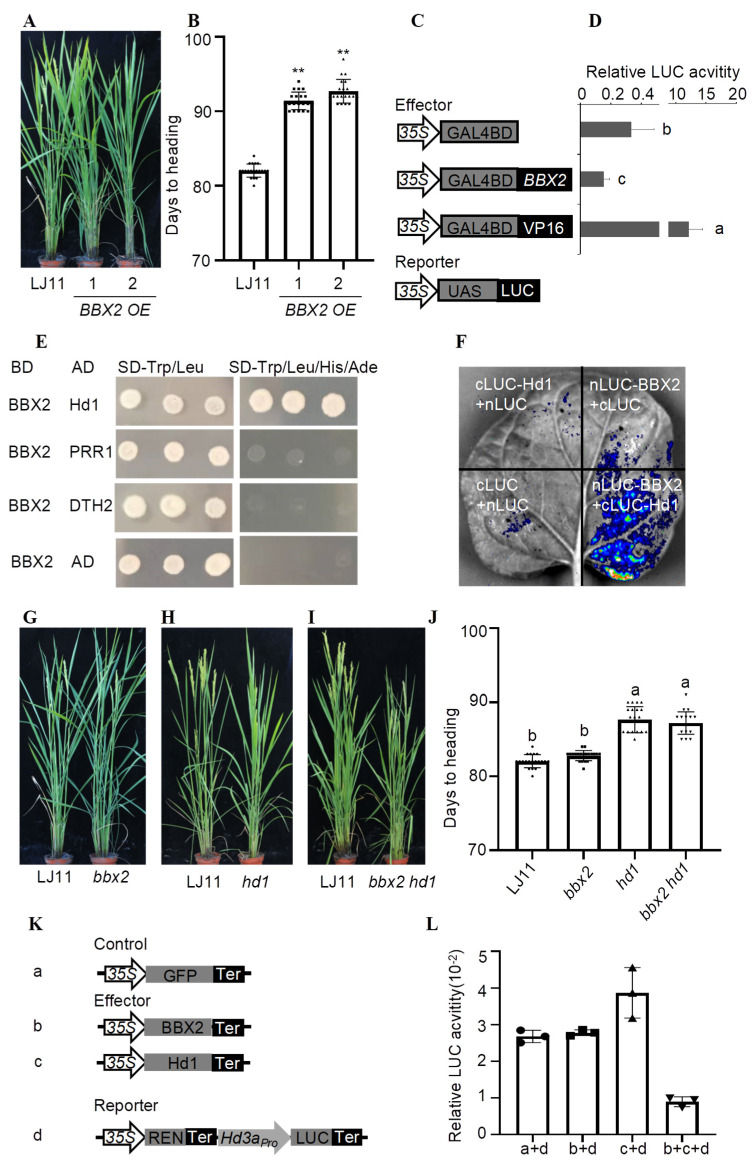
*OsBBX2* delays flowering by repressing *Hd3a* expression. (**A**) Representative image of LJ11 and *BBX OE* plants grown under LD conditions at the heading stage. (**B**) Flowering time of LJ11 and *BBX OE* under LD conditions. Data are means ± standard error (SE; *n* = 20). *p* values were calculated by Student’s *t* test compared to LJ11; **: *p* < 0.01. (**C**) Schematic diagrams of the reporter plasmids used in the rice protoplast transient assay. REN, Renilla luciferase; LUC, firefly luciferase. (**D**) The LUC activity in rice protoplasts with indicated reporter plasmids. Data are means ± SE (*n* = 3). Statistically significant differences are indicated by different lowercase letters (*p* < 0.05, one-way ANOVA with Tukey’s significant difference test). (**E**) The yeast two-hybrid assay showed that BBX2 interacts with Hd1. Yeast grew at 30 °C for 3 days. Empty vectors were used as the negative controls. AD, activation domain. BD, binding domain. (**F**) The LCI assay of the *BBX2* interaction with *Hd1* in *N. benthamiana* leaves. The co-transformation of cLUC-*Hd1* and nLUC-*BBX2* led to the re-constitution of the LUC signal, whereas no signal was detected when cLUC-*Hd1* and nLUC, cLUC and nLUC-*BBX2*, and cLUC and nLUC were co-expressed. In each experiment, at least five independent *N. benthamiana* leaves were infiltrated and analyzed. (**G**–**I**) A representative image of LJ11, *bbx2* (**G**), *hd1* (**H**), and *bbx2 hd1* (**I**) mutants grown under the LD condition at the heading stage. (**J**) The flowering time of LJ11, *bbx2*, *hd1*, and *bbx2 hd1* mutants under LD conditions. Data are means ± standard error (SE; *n* = 20). Statistically significant differences are indicated by different lowercase letters (*p* < 0.05, one-way ANOVA with Tukey’s significant difference test). (**K**) Schematic diagrams of the reporter plasmids used in the rice protoplast transient assay. *35S_Pro_:GFP* was used as the control and *35S_Pro_:BBX2*, *35S_Pro_:Hd1*, and *Hd3a_Pro_:LUC* were used as the effectors and reporters. (**L**) Relative LUC activity expressed with reporters and effectors. The expression level of Renilla (REN) was used as an internal control. The LUC/REN ratio represents the relative activity of the *Hd3a* promoter. Data are shown as means ± SE (*n* = 3). Statistically significant differences are indicated by different lowercase letters (*p* < 0.05, one-way ANOVA with Tukey’s significant difference test).

## Data Availability

All data generated or analyzed during this study are included in this published article and its Supplementary Materials.

## Supplementary Materials

The following supporting information can be downloaded at https://www.mdpi.com/article/10.3390/plants14010048/s1. Supplemental Figure S1: Characterization of *BBX2 OE* plants in the LJ11 background. Supplemental Figure S2: Expression profiles of *BBX2*. Supplemental Figure S3: Characterization of *bbx2*, *hd1*, and *bbx2 hd1* mutants in LJ11 background. Supplemental Table S1: Primers used in this study. Refs. [24,25] was cited in the Supplementary Materials.
