# Supplementary material for: OsBBX2 Delays Flowering by Repressing Hd3a Expression Under Long-Day Conditions in Rice
Source: Plants (Basel). 2024 Dec 27;14(1):48. doi: 10.3390/plants14010048 (PMC11723354; doi:10.3390/plants14010048)
Supplement: Supplementary file 1 [file plants-14-00048-s001.zip › plants-3359900-supplementary.pdf]

Supplemental data

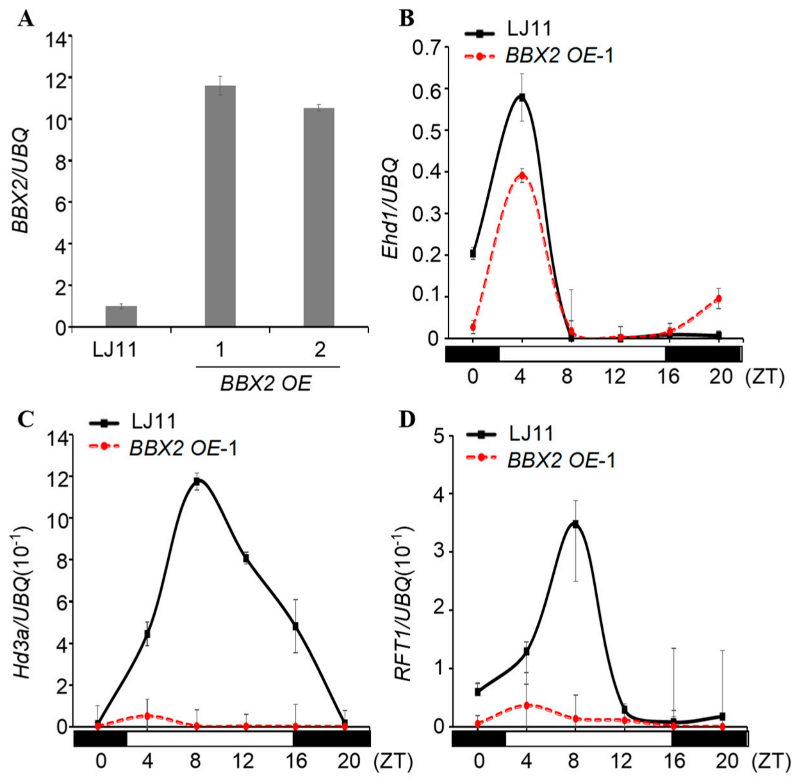

Supplemental Figure S1. Characterization of *BBX2 OE* plants in the LJ11 background.

(A) RT-qPCR analysis of *BBX2* transcription level in *BBX2 OE* and LJ11 plants. The expression level in LJ11 was set as “1”; data are shown as means ± SE (n = 3).

(B-D) Rhythmic expression patterns of *Ehd1* (B), *Hd3a* (C) and *RFT1* (D) in *BBX2 OE* and LJ11 plants under LD conditions. Black and white boxes denote dark and light periods, respectively. Rice *UBIQUITIN* gene was used as the internal control. Data are means ± SE (n = 3). ZT, zeitgeber time.

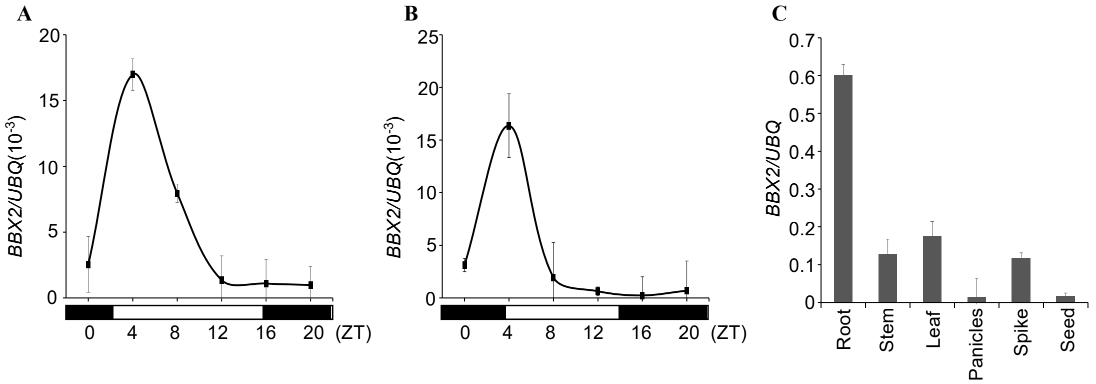

Supplemental Figure S2. Expression profiles of *BBX2*.

(A-B) Rhythmic expression patterns of *BBX2* in LJ11 plants under LD (A) and SD (B) conditions. Black and white boxes denote dark and light periods, respectively. Rice *UBIQUITIN* gene was used as the internal control. Data are means ± SE (n = 3). ZT, zeitgeber time.

(C) RT-qPCR analysis of expression levels of *BBX2* in different tissues. Data are means ± SE (n = 3).

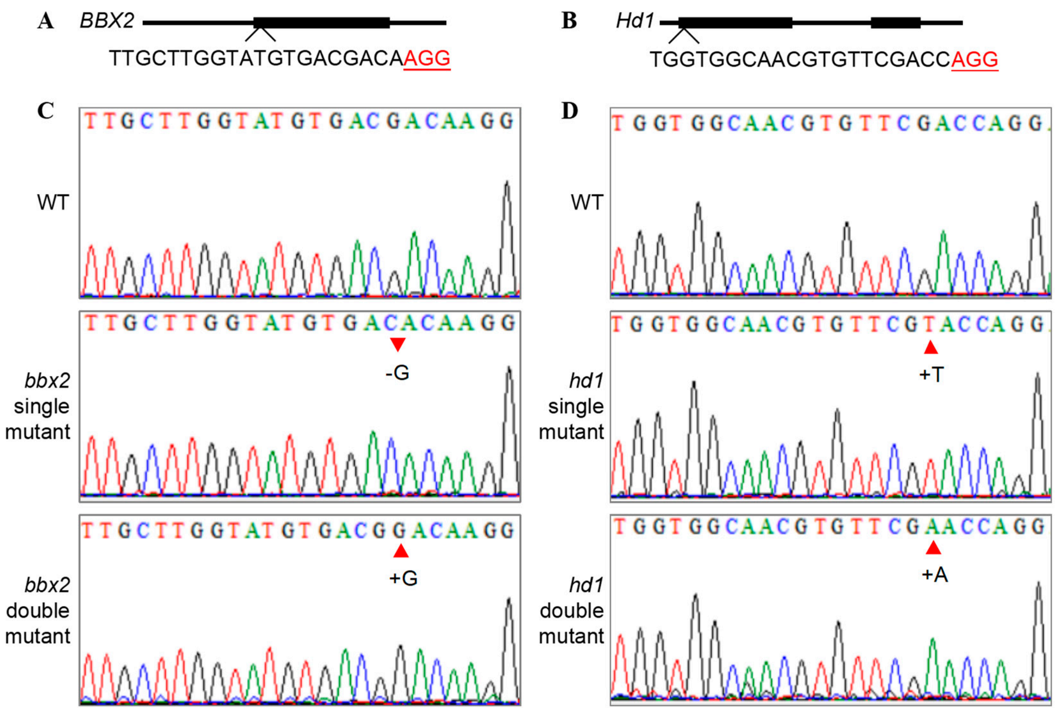

Supplemental Figure S3. Characterization of *bbx2*, *hd1* and *bbx2 hd1* mutant in LJ11 background.

(A-B) Diagram of the genomic region and the sgRNA target site of *BBX2* (A) and *Hd1* (B). The PAM motif (NGG) is shown in red.

(C-D) Sequencing chromatograms of *bbx2*, *hd1* and *bbx2 hd1* mutant in LJ11; Triangle denotes insertion and inverted triangle represents deletion as indicated.

Supplemental Table S1 Primers used in this study.

Primer name	Primer sequence (5'-3')
Hpt-F	TGCGCCCAAGCTGCATCAT
Hpt-R	TGAACTCACCGCGACGTCTGT
BBX2-QRT-F	CCGCGTTTCTTTGCTTGGTA
BBX2-QRT-R	TCCTCATCCACCACCTCAAC
Ehd1-QRT-F	ATGGCTTCAAGTGGAGACAC
Ehd1-QRT-R	ATATTGATGGAGGATGACCG
Hd3a-QRT-F	GCTCACTATCATCATCCAGCATG
Hd3a-QRT-R	CCTTGCTCAGCTATTTAATTGCATAA
RFT1-QRT-F	TGACCTAGATTCAAAGTCTAATCCTT
RFT1-QRT-R	TGCCGGCCATGTCAAATTAATAAC
Ubi-F	ACCACTTCGACCGCCACTACT
Ubi-R	ACGCCTAAGCCTGCTGGTT
Hd1-daba-F	GTGACTTTCCCCTCCCTAGCTCC
Hd1-daba-R	TCTCCTCCTCCTCCTCCTTCG
BBX2-daba-F	GACTGGTGTCCCTGTTTTGC
BBX2-daba-R	TGCCCACAAGTTTCGCATTT
1390-BBX2-F	GTTACTTCTGCACTAGGTACCATGGAGGTCGGCAACGGCAA
1390-BBX2-R	TCTTAGAATTCCCGGGGATCCGGAGCACTCGGCCCAGCCC
nLUC-BBX2-F	ACGGGGGACGAGCTCGGTACCATGGAGGTCGGCAACGGCAA
nLUC-BBX2-R	CGCGTACGAGATCTGGTCGACGGAGCACTCGGCCCAGCCC
cLUC-Hd1-F	TACGCGTCCCGGGGCGGTACCATGAATTATAATTTTGGTGGCA

cLUC-Hd1-R	ACGAAAGCTCTGCAGGTCGACGAACCATGGAACAGTACCATA
BD-BBX2-F	ATGGCCATGGAGGCCGAATTCATGGAGGTCGGCAACGGCAA
BD-BBX2-R	CTAGTTATGCGGCCGCTGCAGGGAGCACTCGGCCCAGCCC
AD-DTH2-F	GCCATGGAGGCCAGTGAATTCATGCTAATAAGCCATCCCAT
AD-DTH2-R	ACGATTCATCTGCAGCTCGAGGTAGCTTCTTGTTTGGCTCA
AD-Hd1-F	GCCATGGAGGCCAGTGAATTCATGAATTATAATTTTGGTGGCA
AD-Hd1-R	ACGATTCATCTGCAGCTCGAGGAACCATGGAACAGTACCATA
AD-PRR1-F	GCCATGGAGGCCAGTGAATTCATGGTGGGCGCCGGCGAG
AD-PRR1-R	ACGATTCATCTGCAGCTCGAGCTCTGGAGAAGAAACCATCT
BBX2-U3-LP	GGCATTGCAACCTGTAATGGAGGT
BBX2-U3-RP	AAACACCTCCATTACAGGTTGCAA
BBX2-U6a-LP	GCCGTTGCTTGGTATGTGACGACA
BBX2-U6a-RP	AAACTGTCGTCACATACCAAGCAA
Hd1-U6b-LP	GTTGTGGTGGCAACGTGTTCGACC
Hd1-U6b-RP	AAACGGTCGAACACGTTGCCACCA
PRT107-BBX2-F	CGCTCTAGAACTAGTGGATCCATGGAGGTCGGCAACGGCAA
PRT107-BBX2-R	TTTGCGGAGTACCCGGGTACCGGAGCACTCGGCCCAGCCC
1390-Hd1-F	GTTACTTCTGCACTAGGTACCATGGCCATGGAGGCCGAATTC
1390-Hd1-R	TCTTAGAATTCCCGGGGATCCCTAGTTATGCGGCCGCTGCAG
PGreen-Hd3apro-F	GGGCCCCCCCTCGAGGTCGACTCAATCACCAGCTAATTGGC
PGreen-Hd3apro-R	CGCTCTAGAACTAGTGGATCCCGATCTTGCAAAAAACCCTG

Materials and methods

Rice materials and growth conditions

Longjing11 (LJ11) cultivar was used to create *BBX OE* and *bbx2*, *hd1* and *bbx2 hd1* mutant by Agrobacterium-mediated co-cultivation transformation experiments, as described previously (Tian et al., 2015). Plants were grown in Harbin (45°N) with long day (14 h light/10 h dark)

condition in the paddy field. Heading dates were recorded from sowing to the appearance of the first panicle.

Generation of transgenic plant and mutant

To generate the *BBX2 OE* transgenic plants, we downloaded the CDS coding sequence of BBX2 from the website (http://rice.uga.edu/) and cloned from the rice variety *Nipponbare*, then inserted into the pC1390U vector to generate the overexpression constructs driving by *Ubiquitin* promoter. To generate *bbx2*, *hd1* and *bbx2 hd1* mutant, gene-specific sequences for the corresponding genes were ligated into the CRISPR/Cas9 binary vectors *pYLCRISPR/Cas9Pubi-H* (Ma et al. 2015). The primers were listed in Supplemental table 1. All constructs were introduced into *Agrobacterium tumefaciens* strain EHA105 and transformed into LJ11.

Gene expression analysis

For expression level of *BBX2*, *Ehd1*, *Hd3a*, and *RFT1*, LJ11 and *BBX OE* transgenic plants were grown for 40 days under LD or SD conditions, and leaf samples were collected. Total RNA was extracted using TRIzol (Invitrogen). cDNA was synthesized from 1.5 µg of total RNA using SuperscriptII Reverse Transcriptase (Invitrogen). Real-time PCR was performed with SYBR Green PCR master mix (TransGen). Data were collected using QuantStudio™ 5 System. All expressions were normalized against the *Ubiquitin* gene (Os01g0328400). The primers used are listed at Supplemental Table 1.

Protoplast transient transformation assays

For the transient transformation assays, the coding regions of BBX2 and Hd1 was ligated into the pRT107 vector to generate the $35S_{Pro}$*:BBX2* and $35S_{Pro}$*:Hd1* used as the effector. The 2051 bp promoter of *Hd3a* was cloned into *pGreenII0800-LUC* as the reporter. Primers used for these constructs are listed in Supplemental Table 1. Rice protoplasts were isolated from stem and sheath tissues of young LJ11 seedlings. Firefly LUC and REN activities were measured with a Dual-Luciferase reporter assay kit (Beyotime, RG027) using a GloMax 20/20 luminometer (Promega). The LUC activity was normalized to REN activity and LUC/REN ratios were calculated. Values are means ± SE of three biological repeats.

LUC complementation imaging (LCI) assays

The coding region of *BBX2* and *Hd1* was ligated into nLUC or cLUC of *pCAMBIA1300* vector. *Agrobacteria* harboring different combination of constructs were coinfiltrated into *N. benthamiana*, and the infiltrated leaves were analyzed for LUC activity using Chemiluminescence imaging (Tanon 5200) after 48 h infiltration.

Yeast two-hybrid assay

In order to detect whether BBX2 could physically interact with Hd1, PRR1 and DTH2. The coding region of BBX2 was ligated into pGBKT7 and Hd1, PRR1 and DTH2 was ligated into pGADT7 vector. Four combinations of constructs were transformed into yeast Y2H strain, and the interaction between them was screened in SD-Trp/Leu/His/Ade deficient medium.

Tian, X., Wang, Z., Li, X., Lv, T., Liu, H., Wang, L., Niu, H., and Bu, Q. (2015). Characterization and Functional Analysis of Pyrabactin Resistance-Like Abscisic Acid Receptor Family in Rice. Rice 8, 28.

Ma, X.L., Zhang, Q.Y., Zhu, Q.L., Liu, W., Chen, Y., Qiu, R., Wang, B., Yang, Z.F., Li, H.Y., Lin, Y.R., Xie, Y.Y., Shen, R.X., Chen, S.F., Wang, Z., Chen, Y.L., Guo, J.X., Chen, L.T., Zhao, X.C., Dong, Z.C., and Liu, Y.G. (2015). A Robust CRISPR/Cas9 System for Convenient, High-Efficiency Multiplex Genome Editing in Monocot and Dicot Plants. Molecular Plant 8, 1274-1284.
